# Osteoporosis: From Molecular Mechanisms to Therapies 2.0

**DOI:** 10.3390/ijms21218005

**Published:** 2020-10-28

**Authors:** Chih-Hsin Tang

**Affiliations:** 1 Department of Pharmacology, School of Medicine, China Medical University, Taichung 404, Taiwan; chtang@mail.cmu.edu.tw; Tel.: +(886)-22052121 (ext. 7726); 2 Chinese Medicine Research Center, China Medical University, Taichung 404, Taiwan; 3 Department of Biotechnology, College of Health Science, Asia University, Taichung 41354, Taiwan

**Keywords:** osteoporosis, treatment, prevention, molecular mechanisms, signaling pathway

## Abstract

Osteoporosis is a common skeletal disorder, occurring as a result of an imbalance between bone resorption and bone formation, with bone breakdown exceeding bone building. Bone resorption inhibitors, e.g., bisphosphonates, have been designed to treat osteoporosis. Teriparatide, an anabolic agent, stimulates bone formation and corrects the characteristic changes in the trabecular microarchitecture. However, these drugs are associated with significant side effects. It is therefore crucial that we continue to research the pathogenesis of osteoporosis and seek novel modes of therapy. This editorial summarizes and discusses the themes of the ten articles published in our Special Issue “Osteoporosis: From Molecular Mechanisms to Therapies 2.0”, a continuation of our 2019 Special Issue "Osteoporosis: From Molecular Mechanisms to Therapies" (https://www.mdpi.com/journal/ijms/special_issues/osteoporosis_ijms). These Special Issues detail important global scientific findings that contribute to our current understanding of osteoporosis.

Osteoporosis is a common skeletal disorder, occurring as a result of an imbalance between bone resorption and bone formation, with bone breakdown exceeding bone building. Bone resorption inhibitors, e.g., bisphosphonates, have been designed to treat osteoporosis. Teriparatide, an anabolic agent, stimulates bone formation and corrects the characteristic changes in the trabecular microarchitecture [1]. Two antibody drugs, denosumab and romosozumab, are targeted to receptor activator of nuclear factor kappa-B (RANKL) and sclerostin show promise in the treatment of osteoporosis [2]. However, these drugs are associated with significant side effects. It is therefore crucial that we continue to research the pathogenesis of osteoporosis and seek novel modes of therapy.

Our call for papers for this Special Issue, *Osteoporosis: From Molecular Mechanisms to Therapies 2.0*, prompted the submission of several articles, all of which were subjected to rigorous peer review. The 10 that satisfied our inclusion criteria for this issue include six papers describing (i) the therapeutic effects of novel agents designed to prevent bone loss caused by prolonged glucocorticoid therapy or diabetes, (ii) the molecular mechanisms of osteoporosis and (iii) in vivo evidence from new treatments showing potential in the prevention and treatment of osteoporosis. We also include four reviews that summarize recent findings on molecular mechanisms of osteoporosis and potential pharmacological options for the management of osteoporosis. All 10 articles are discussed below.

(i) The therapeutic effects of novel agents designed to prevent bone loss caused by prolonged glucocorticoid therapy or diabetes. Preclinical evidence from Asri and colleagues suggests that aqueous *Piper sarmentosum* leaf extract may effectively prevent bone loss in patients on prolonged glucocorticoid therapy [3]. Zheng and colleagues discuss their study evidence showing that chondroitin sulfate protects against diabetic osteoporosis using a streptozotocin-induced diabetic rat model [4].

(ii) The molecular mechanisms of osteoporosis. Durbano and colleagues have identified aberrant signaling within the bone morphogenetic protein (BMP) signaling pathway in patients with osteoporosis [5]. The study researchers hypothesize the reasons for this signaling disparity and they call for further analysis into this BMP signaling cascade, as this might reveal opportunities to design more effective therapeutics [5]. Liu et al., indicated that the migration and metastasis of abnormal osteoblasts (osteosarcomas) is regulated by CXCL3/CXCR5 interaction [6].

(iii) In vivo evidence from new treatments showing potential in the prevention and treatment of osteoporosis. Sequeira and colleagues discuss how their novel peptide, CK2.3, demonstrated not only anabolic and antiresorptive effects on bone in ovariectomized rats, but also improved fracture resistance [7]. The paper submitted by Ahn and colleagues describes promising therapeutic effects of tauroursodeoxycholic acid on bone in a mouse model of osteoporosis [8].

The review articles discuss the basic molecular mechanisms of bone remodeling, investigational treatments, and potential therapeutic approaches [9], as well as evidence from preclinical investigations showing that the phytochemical lycopene may prevent postmenopausal bone loss [10] and that the flavonoid quercetin has bone-protective qualities [11]. The last review article describes useful interventions and treatment strategies that can be applied in the management of patients with mineral bone disorder associated with chronic kidney disease [12].

We hope that this Special Issue will be of great interest for researchers who are seeking to develop novel osteoporosis prevention and treatment strategies.
